# Order Through Disorder: The Characteristic Variability of Systems

**DOI:** 10.3389/fcell.2020.00186

**Published:** 2020-03-20

**Authors:** Yaron Ilan

**Affiliations:** Department of Medicine, Hadassah-Hebrew University Medical Center, Jerusalem, Israel

**Keywords:** randomness, quantum biology, biological variability, evolution, system biology

## Abstract

Randomness characterizes many processes in nature, and therefore its importance cannot be overstated. In the present study, we investigate examples of randomness found in various fields, to underlie its fundamental processes. The fields we address include physics, chemistry, biology (biological systems from genes to whole organs), medicine, and environmental science. Through the chosen examples, we explore the seemingly paradoxical nature of life and demonstrate that randomness is preferred under specific conditions. Furthermore, under certain conditions, promoting or making use of variability-associated parameters may be necessary for improving the function of processes and systems.

## Introduction

Randomness is observed in a variety of natural processes (Rajdl et al., 2017). Its existence and, more importantly, role in various developmental processes has been determined and documented (Longo and Bertolotti, 2017). Chance/Darwinian selection principles are partially dependent on probabilistic laws that refer to random events, while molecular biology is based on deterministic premises that regard randomness as “noise.” Recent data suggested that an intrinsic stochastic dimension of intracellular pathways (Heams, 2014; Ramsey, 2016). The concept of randomness is used in a variety of ambiguously connected ways in the scientific literature. The term “randomness” is used in some studies as a substitute of disorder in the thermodynamic sense. This is exemplified by its use in nucleotide substitutions in DNA sequence (Zhang and Gerstein, 2003). Under different settings, “random” is used for meaning of variation. This is demonstrated when referring to the evolving of ancestral DNA sequence in different forms in a population of organisms of the same species (Birky et al., 2010). The term “randomness” is also being used when referring to the complexity of biological systems (Varella, 2018; El-Haj et al., 2019; Ilan,2019b,c,d). In addition, this term can sometimes be used for describing noisy or stochastic behavior of systems, emphasizing some type of unpredictability of these structures (Trewavas, 2006; Tsimring, 2014; El-Haj et al., 2019; Ilan, 2020). Randomness also has some evolutionary implications, where variation and probability are linked with adaptation (Latta, 2008, 2010).

The aim of the present paper is to provide some examples where the term “randomness” is used and may play some type of causality in natural processes. Our study provides examples of the natural tendency toward randomness observed across various systems under certain conditions. We propose the concept of using randomness for improving processes and functions.

### Randomness in Biology

Randomness, defined as the apparent lack of pattern or predictability in events, characterizing many biological systems and, moreover, fundamental for their correct function (Ilan,2019a,b,c). A biological system can manifest contradictory interrelations with randomness. Corroborating that apparent random events are truly random is difficult. The distinction between purely random and partially random variation is important for understanding the nature and evolution of biological systems. Inherent bias prevents total randomness. Completely unbiased random changes in a sustainable complex system are impossible, by virtue of constraints imposed by the intrinsic organization in the system (Warren et al., 2018). Models of kinetic reaction-diffusion equations are used to describe microscopic biological populations dynamics (Cordier, 2016).

In contrast to the randomness described in physics, the range of possibilities in biology is not provided, but rather randomly co-constituted (Longo et al., 2012, 2015; Buiatti and Longo, 2013; Montevil et al., 2016). Randomness is a feature of evolutionary biology and genetics (Heams, 2014). Biological objects are characteristically diverse, display randomness, and may undergo irregular and unpredictable changes (Montevil et al., 2016). Darwin suggested that random variation generates new living forms of life. He proposed that variation, uncorrelated with the later selected function, was the engine of evolution by natural selection. Organisms have evolved to adapt to variability at the molecular level. The successful evolution of microorganisms can be partially attributed to their ability to adapt to unpredictable changes in their environment. Alterations range from deterministic mechanisms to randomness at the level of cellular pathways. Neo-Darwinism expressed “blind chance” as an origin of variation. Furthermore, spontaneous and induced mechanisms of phenotypic adaptation indicate the role of chance and constraints in microbial phenotypic adaptation (van Boxtel et al., 2017). A distinction is made between selection which acts on any phenotypic difference, and the response to selection which happens when the phenotypic differences are heritable. It differentiates natural selection associated with filtering of variation, from the development of new variants due to selection (Latta, 2010; Hardy et al., 2018). Selection can be based on genetic variation or on conditionally natural variation (Hermisson and Wagner, 2004; Ledon-Rettig et al., 2014; Paaby and Rockman, 2014).

Organisms are specific objects which are different from each other and undergo unpredictable changes. Variability in biological systems can be viewed as a contributing modality of their function (Clarke and Crame, 2010; Farahpour et al., 2018). Randomness can be considered as a property of causal processes, contributing to complex system function (Ilan,2019e; Kenig and Ilan, 2019; Khoury and Ilan, 2019). Random genetic drift is accepted as the random fluctuations in the numbers of gene variants in a population (Lynch et al., 2016). Noisy or stochastic behavior of systems may be part of their dynamic behavior in response to various internal and external triggers. It describes these type of systems as stochastic dynamical systems versus being rigid, non-adaptable, and non-responsive to triggers (Freeman et al., 2001).

Chaos refers to an apparent disorder arising in deterministic systems that are highly sensitive to initial conditions. In the frame of dynamical systems theory, with a strong dependence on initial conditions and randomness, chaos may increase the unpredictability of evolution, but it may not entirely undermine its predictability (Rego-Costa et al., 2018). In evolution, chaos may increase under appropriate conditions due to competitive interactions. Populations develop in response to changing environments, which affect the evolution of structures prone to chaos. Environmental forces can lower the probability of chaos via the reduction of chaotic oscillations (Rego-Costa et al., 2018).

Cases of order within the human body which are associated with the development of diseases were described; the concept is based on the notion that natural diversity is defined by discreteness and order (Alberch, 1989). Current schemes in biological systems suffer from a lack of precise definitions. The term “heterochrony” refers to both a developmental process and an evolutionary pattern, which are microevolutionary processes of adaptation in local populations under selection, respectively, a macroevolutionary pattern based on undefined internal laws of form (Alberch and Blanco, 1996). It was suggested to reduce the timing and focus dependence of heterochrony on the organization of sequences of developmental events in ontogeny. Selection during ontogeny can result in the evolution of the developmental process (Blanco and Alberch, 1992). Limb morphology was proposed to vary during evolution, implying that developmental constraints impact the morphological evolution development (Oster et al., 1988). The association between ontogeny and phylogeny involves variable ontogenetic patterns and complex biological systems. Molecular heterochrony requires a linear or strictly hierarchical structure of gene regulation of development, along with isomorphism between genetic mutations and morphological changes (Alberch and Blanco, 1996).

Two philosophical approaches explained the order in nature. Classical neo-Darwinism, also referred to as the “externalist” conceptual position, is based on accepting natural selection as the ordering method in evolution. Properties of the physical and biotic environments determine the selective pressures which drive the final natural selection made. Under these conditions, the discreteness and order of diversity reflect the topography of the adaptive landscape. The second position, referred to as the “internalist” approach, attributes the order in nature to emergent properties of generative rules. It was proposed that “genes and development cannot be dissociated as different levels of interaction” (Alberch, 1989). Associations between evolution, development and pathologies underlie biological system development and macroevolution (Diogo et al., 2017). These were suggested as tools for understanding human anomalies and variations, homeotic transformations, and muscle-skeleton associations in limb and facial muscles. Also, they were proposed to explain developmental constraints linked with birth defects, human evolution, trisomies 13, 18, 21, and other disorders (Diogo et al., 2015).

The central dogma of molecular biology, that functional order at a high level is a direct result of order at the molecular level, was proposed by Crick and Watson. They acknowledged Schrödinger’s work influence in their thinking (Cobb, 2017; Noble, 2017). Kupiec (2014) already described two problems arising from this approach. The first was that the central dogma refers only to the sequence information that passes from DNA to proteins. While targeted molecular mechanisms regulate the function, there is no guarantee that the functionality will be evident at the molecular level. This happens because it requires multiple correlations between patterns of binding and functional processes at a higher level, to identify the functionality involved. Without that correlation, the binding patterns appear random. The second problem is that, as Schrödinger argued as well for physics, there is no way in which the molecules in an organism can avoid stochasticity (Kupiec, 2009; Deaton and Bird, 2011; Corre et al., 2014; Noble, 2017). Competitive advantages and aggressiveness of certain species were described in various biological systems as contributing factors for selection (Ravinski, 1982; Rawinski and Maleclri, 1984).

### Randomness and Biodiversity

The environment is spatially heterogeneous, and this attribute impacts the efficiency of minute dynamics at a local level (Ornstein, 1989). It prevents the synchronization of locally evolving oscillations, leading to a state in which one form of randomness lessens the effects of other forms of randomness in the system (Szolnoki and Perc, 2016). The fact that global oscillations are rarely noted in biological systems was proposed to partially underlie biodiversity (Buiatti and Longo, 2013).

### Randomness in Biological Functions

Randomness does not necessarily imply a lack of function. In fact, it is used by organisms to generate functionality (Noble, 2017). This idea contradicts Schrödinger’s assertion that phenotypic order is generated from the molecular level, which is the underlying dogma of molecular biology. Living systems perform in a compound and, sometimes, irregular manner, which is hard to predict. For example, in many biological systems, cells are crowded within spatially heterogeneous spaces, associating with biomolecules whose activities are controlled by random forces. This form of randomness leads to an array of precisely coordinated biological functions, such as transcription, translation, ribosome biogenesis, chromosome replication, and metabolism, as well as complex behaviors (Bertolaso et al., 2015; Fels, 2018; Ilan,2019c,e; Ilan-Ber and Ilan, 2019).

Models using linear and non-linear differential equations can capture the compound biochemical and genetic responses of tissues and cells. For cases where cellular performances are stochastic, such as during single-cell responses, the addition of randomness has been shown to improve distinct deterministic models (Selvarajoo, 2018). Thus, randomness can be useful for modeling systems and engineered control of systems (Brandao et al., 2016). In particular, stochasticity can be augmented with atomic-scale molecular modeling approaches (Earnest et al., 2018).

Randomness is one of the methods which can be useful to generate order. For example, this is evident in the biological self-organization seen in dividing cells, wherein microtubules associate with mitotic kinetochores in a conventional manner (Ilan,2019c,e; Ilan-Ber and Ilan, 2019). Each sister kinetochore forms microtubule accessories to a single spindle pole. The order by which this process unfolds differs from cell to cell, in accordance with the different locations of kinetochores and spindle poles, as well as the randomness of the original microtubule attachments. Despite this, the outcome is reproducible with high fidelity due to the process of chromosome segregation, which avoids improper microtubule attachments (Lampson and Grishchuk, 2017). Thus, a certain degree of order or control is achieved through an initial state of stochasticity.

### Genes Proteins and Randomness

Over the last two decades, it was proposed that gene expression may involve stochastic processes (Gandrillon et al., 2012; Boettiger, 2013). The genetic circuits controlling cellular functions are subject to stochastic fluctuations, “noise,” which play an important role in key cellular activities. For example, noise functions in microbial and eukaryotic cells, regulating gene expression and contributing to differentiation strategies that function across cell populations (Eldar and Elowitz, 2010).

Isogenic cells cultured in a similar environment display random variations in gene expression. The regulation of gene expression includes reactions between a finite number of molecules, which result in randomness of the gene expression dynamics (Phillips et al., 2017). Gene expression activity is heterogeneous, while molecular steps and mechanisms of transcription and translation involve randomness at many levels. For example, alterations in the mitochondrial content of each cell contribute to heterogeneity in gene yields, as well as phenotypic diversity (Guantes et al., 2016).

The second law of thermodynamics states that the entropy of an isolated system can only increase. With the entropy defining the degree of randomness, disorder is expected to increase over time. Concerning gene expression, it is unclear whether sequence randomness varies over time and whether it does so consistently with the second law of thermodynamics. In a recent study, the sequence randomness was investigated based on a collection of statistical tests and by evaluating the variability of coding sequences of *Escherichia coli*. Ancient core/essential genes were found to be more random than the less ancient specific/non-essential genes. Consistent with the second law, sequence randomness increased over time. Moreover, this change led to increased randomness of GC content and longer sequence length (Wang G. et al., 2016).

Chromosomes occupy distinct domains, “territories,” in the cell nucleus. Models of non-random nuclear chromosome organization imply a role for this platform in their function and in the generation of genome stability (Parada and Misteli, 2002). Chromosomal regions are modified by histones, which are responsible for the randomness of nucleosome positions. In contrast to equidistant nucleosomes, randomly spaced nucleosomes exhibit heritable bistability (Tan et al., 2016). Direct recruitment of transcription activation domains in gene-specific activators and basal transcriptional machinery components show an increased level of sequence randomness. The sequence randomness and intrinsic structural disorder of these domains are essential for temporary changing associations with promoter nucleosomes, which activate promoter nucleosome translocation, followed by gene stimulation (Erkina and Erkine, 2016).

Single-cell analysis revealed wide stochasticity of gene expression, with individual genes undergoing cycles of activity bursts and inactivity periods. The range of variability of genome organization was suggested to influence the genome function. Specific chromatin-chromatin interactions are present in a small fraction of cells, and the genome organization at the level of individual alleles in the same nucleus differs considerably. The high degree of variability is characterized by fluidity of the chromatin domains, and by a high degree of variability in the genomic positions of the boundaries between chromatin domains from one allele to another. The highly variable nature of genome architecture points to a high degree of intrinsic noise in genome organization, in line with the observed stochasticity in gene expression. These may be required for a proper function of the system (Finn and Misteli, 2019).

Incomplete penetrance occurs when not all the carriers carrying mutations that cause genetic disorders develop the associated disease. There are cases in which only a small fraction of a population of organisms harboring a mutation, at a particular genetic locus, develops the phenotype associated with the mutation. Incomplete penetrance is also found in genetically identical populations under controlled, stable conditions. This phenomenon indicates the presence of a mechanism through which diversity is generated even among genetically identical individuals, and is required for proper outcome (Raj et al., 2010; Streit and Sommer, 2010).

Evolutionary processes modify genetic variation in a focal species, impacting the interactions with other species and the intraspecific genetic variation (Genung et al., 2010). In immunology, the clonal selection theory explains the functions of cells of the immune system in response to specific antigens invading the body via the generation of diversity of antibody specificity. This was postulated to account for the absence of immunological response to self-constituents and the related phenomena of immunological tolerance (Burnet, 1962; Burnet and Holmes, 1962). The theory explains the self-non-self-discrimination despite the lack of information regarding the cellular and molecular basis of the immune response (Klinman, 1996).

Gene transcription may involve exceedingly stochastic processes. The mRNA copy number of a given gene varies across cells and is time-dependent. A model for gene transcription in cells with variability in intervals, which is both stochastic and deterministic, was developed. The rates expressed the contributions of different effects of the environment (Dattani and Barahona, 2017). Bayesian regression models can account for the randomness of genotypes across population genome-wide predictions. These models are diverse and correlate the marker effects and allelic frequencies within different populations (Martinez et al., 2017). However, the current models used for genome-wide prediction overlook the randomness of genotypes. Hierarchical Bayesian models overcome this limitation by displaying genotypes as random variables enabling population-specific effects for separate markers. Further, these models also account for the heterogeneity of allelic frequencies (Martinez et al., 2017).

Protein biosynthesis processes was described by using deterministic conceptual models (Cobb, 2017). The mechanistic understanding of these processes has led to their refinement and newer models were developed. The method by which proteins are made, similar to other systems, may comprise deterministic approach, and under certain conditions some degree of randomness (Choi et al., 2010; Phillips et al., 2016). In this case, the randomness can arise from the distinct stochastic character of chemical reactivity (“intrinsic” noise), the variability among cells in the number of molecules involved in gene expression, or the intervals of cell-cycle events (“extrinsic” noise), such as DNA replication and cell division (Longo and Hasty, 2006; Soltani et al., 2016).

Protein copy numbers vary among cells. Since protein half-lives are equivalent to the length of the cell cycle, the agreement is that randomness in cell division times can generate additional intercellular variability in protein levels. Assessment of the noise at the level of the proteins – at times when the cell-cycle period follows probability distributions – showed that it could have constituents arising from stochastic expression, separating asymmetry at the time of cell division, and random cell division events. The extrinsic noise generated during random division periods of the cells impacts the number of proteins and the intrinsic noise components (Soltani et al., 2016).

### The Randomness Parameter in Enzymatic Reactions

Temporal fluctuations in the catalytic rates for single-enzyme reactions which change between two active states were demonstrated. Further, the randomness parameter may be useful in models in which conformational distinctions occur between two free enzyme conformers. Enzymatic variations produce a non-Michaelis–Menten equation and are associated with dynamic cooperativity. The randomness parameter reflects the dynamic disorder of a structure, which converges toward unity at increased substrates concentrations (Singh and Chaudhury, 2017). The randomness parameter did not equal unity at intermediate to high substrate amounts, when evaluated for the activity of a single enzyme in the presence of multiple substrates. This suggests the presence of numerous rate-limiting steps in the reaction and shifts between the free enzyme and the enzyme-substrate complexes (Singh and Chaudhury, 2018). Another example is the enzyme-catalyzed synthesis of poly(3-hydroxybutyrate-co-butylene succinate) copolyesters, which has shown random macromolecular behaviors correlated with reaction time, oligomer chain length and selected solvent (Debuissy et al., 2016).

The traditional theory of enzymatic inhibition uses a deterministic approach to define quantitatively the impact of inhibitors on enzymatic reactions. However, the presence of intrinsic randomness changes the concept of modes of inhibition. Stochastic variations at the single-enzyme level can lead to a state where inhibitors function as activators. For example, molecules that inhibit enzymes when substrate concentrations increase may cause a non-monotonic dose-response when concentrations are reduced (Robin et al., 2018).

### Randomness: From Microbes to Plants and Animals

A cell exposed to a new environment shows a transcriptional response that is controlled by its history. Also, randomness contributes to the response to a new environment. For yeast cells, the change in their speed depends on prior nutrition availability. For example, cellular memory of long-term glucose exposure reduces GAL activation with a high degree of variability among cells, while exposure to other nutrients could promote uniform responses (Stockwell and Rifkin, 2017). A yeast polarization model have shown that fuzzy stochastic nets are a suitable choice for modeling systems with uncertain information (Liu et al., 2016). A measure of neural spike randomness, an entropy factor, was proposed based on the Shannon entropy of the proportion of spikes in a time interval. Entropy factors were assessed from experimental data of spontaneous activity in the macaque primary visual cortex and compared to theoretical values, evaluated from models of renewal process. An increased spike count variability was associated with an increase in predictability, as indicated by the entropy factor (Rajdl et al., 2017).

Humans assimilate intuitive statistics with other cognitive domains, while evaluating the randomness of an event. Chimpanzees, like humans, have a random sampling assumption that can be overruled under the proper conditions, and can use mental state data to assess whether this is required. Chimpanzees select based on proportional data while lacking information about the experimenters’ choices and biases for specific food types they tended to draw blindly. However, when biased experimenters gained visual access, they disregarded statistical data and selected based on their biases (Eckert et al., 2018).

Catastrophic genome rearrangements can occur spontaneously and then stabilize during plant somatic growth, leading to the generation of new phenotypes of grape (*Vitis vinifera*). Color somatic variants are associated with deletion events of uncertain origin. Validated clustered breakpoints, associated with intra- and inter-chromosomal translocations among three linkage groups, flank the deleted fragments in a single copy of each of the affected chromosomes. The randomness of the direction of rearranged fragments aligned with a chromothripsis-like shape, generated after chromosome breaking and illicit rejoining (Carbonell-Bejerano et al., 2017).

Finally, randomness in the motion of animals is relevant to the movement of individuals within animal groups. It promotes the compactness of a herd by enabling the disintegration of small clusters in favor of a more compact structure and, by that, reducing the distances to center for the herd (Ose and Ohmann, 2017).

### Randomness in Physics

Permutation entropy and network entropy are distinct entropy types, described in epsilon-recurrence networks and used for determining the degree of randomness in the primary dynamics of systems. Both exhibit alterations in the dynamic performance of flow velocity variations. The two types of dynamics, a low-dimensional deterministic chaos in the near field, controlled by the motion of large-scale vortices, and a high-dimensional chaos in the far field, making a turbulent plume, were recognized by a multiscale complexity-entropy causality plane (Takagi et al., 2017).

Randomness leads to cascading failures of spatially interdependent networks. The impact of dependency maps randomness on the toughness of interdependent lattice networks has been investigated. Approximate entropy (ApEn) is employed to reflect the randomness degree of dependency maps (Yuan et al., 2016). The entropy of a given signal-type waveform is considered to be zero. However, it is required to use entropic measures for assessing the magnitude of related data. Fourier-conjugated “total entropy” has been linked to quantum-mechanical probabilistic amplitude functions, as a measure of the information in non-probabilistic real waveforms. This entropy is sensitive to the degree of randomness in a sequence of pulses and can distinguish weak signals (Funkhouser et al., 2016).

Moderate-high-energy inelastic neutron scattering experiments were used to determine the crystalline electric field energy levels in the triangular spin-liquid candidate YbMgGaO. Randomness in the crystalline electric field levels was found to induce the dissemination of effective spin-1/2 g factors, explaining the unparalleled expansion of low-energy magnetic excitations in the fully polarized ferromagnetic phase (Li et al., 2017). In another example, the spatial distribution of separate photons generated by spontaneous parametric down-conversion does not show any spatial mode structure, due to the randomness of their generation, as well as their incoherent characteristics (Diogo et al., 2015).

### Randomness in Chemistry

In chemistry, a random disorder may occur in the presence of strong interactions, contrary to the accepted hypothesis that weak interactions between components cause random disorder.

The lead-based complex perovskite Pb (In_1__/__2_Nb_1__/__2_)O_3_ has an inhomogeneous structure. Phase transitions in its single crystals are ascribed to changes in the degree of randomness with which a specific lattice site is occupied by In and Nb atoms. The dynamic properties of this compound are also dependent on the degree of randomness. It was found that, for a regular distribution of occupied In/Nb sites, the anti-ferroelectric phase is stabilized and the growth of the inhomogeneous structure is suppressed. However, for randomly occupied sites, a fractal structure develops as the temperature decreases, and nanosized ferroelectric domains are shaped (Tsukada et al., 2017).

A sequence of charge-balanced polyampholyte physical hydrogels, obtained by random copolymerization in water, has been shown to display increased toughness, self-healing ability and viscoelasticity. Their function was attributed to dynamic ionic bond development through inter- and intra-chain connections. This randomness resulted in ionic bonds with a large strength range, from strong bonds, which serve as lasting crosslinking, to weak bonds, which can break and re-form reversibly, dispelling energy (Cui et al., 2016).

Hybrid solar cells are fabricated on thick crystalline Silicone thin films. A surface treatment process involving oxygen plasma was applied on cells with Si nanowires, for improving power conversion efficiency. The characteristic randomness of treated cells, in terms of dimensions and spacing, was associated with an improved light-harvesting capability (Wang H. et al., 2016). In a similar example, the lowest thermal conductivity of silicon-germanium bulk alloys was made possible via alloy scattering. The thermal conductivity varied according to the amount of randomness in the spatial organization of the constituent atoms (Lee et al., 2016).

The induction of mechanochemical transformations depends on inserting target molecules, mechanophores, within elastomeric polymer networks. The molecular structure randomness – characteristic to such materials – leads to the variability of the local forces exerted on individual mechanophores (Adhikari and Makarov, 2017).

### Randomness in the Human Body

By employing concepts of complexity, it was proposed that traditional physiology be substituted with fractal physiology, in which variability reflects the health status better than the average (West, 2006; El-Haj et al., 2019; Ilan,2019a,b,c,e,f). It was submitted that variations in heart rate, breathing and walking are much more sensitive to the early effects of disease than their means. For example, initiation and termination of action potentials by cardiomyocytes can be viewed as random events. The origin of randomness is determined by a statistical analysis of the events in question (Song et al., 2017). It has been found that the variability noted in cardiomyocyte action potential measurements originates from different sources of randomness, such as ionic channel conductance (Tixier et al., 2017).

Non-linear parameters defining the chaos, fractal features and complexity of heart sound signals were used to evaluate heart sound signals from patients with chronic heart failure (CHF). These parameters have been found to differ significantly between healthy participants and CHF patients, with the latter showing decreased chaotic characteristics, complexity, and randomness (Yineng and Xingming, 2017).

Multifractality is used for the assessment of physiological variability. Studies on heartbeat time series employed two different diagnostic techniques. The first one evolved from findings that the multifractal spectrum of healthy subjects is broader than the multifractal spectrum of patients with a disease. The second was based on data showing that heartbeat dynamics are a superposition of crucial and uncorrelated Poisson-like events, with patients showing a higher probability of uncorrelated Poisson-like events than healthy subjects. Increasing the magnitude of uncorrelated Poisson-like events held by heartbeats leads to a narrowing of their multifractal spectrum (Bohara et al., 2017).

In a study of two groups of infants, one fed by the gentle co-regulated feeding approach while the other by the usual care approach, the former group presented fewer apneic events and higher respiratory rate, as well as significantly lowers heart rate variability, which is a potential indicator of elevated stress. This indicates that, during co-regulated feeding, infants display increased breathing. No statistically significant differences due to diet – formula versus regular diet – were found in the oxygen saturation, heart rate, bradycardia, or high-frequency power, which are the most useful measures of heart rate variability (Pados et al., 2017).

### Randomness in the Human Brain

In the central nervous system, functional networks are constructed in accordance with the isomorphism between networks, under the assumption that they are deterministic. However, the functional brain networks differ among people from the same population, due to personalized characteristics. Nonetheless, the functional networks of humans from diverse populations can be generated using a similar stochastic process (Fujita et al., 2017). Mathematical modeling of neural activity suggests that the internal and external variability is strikingly similar (Dumont et al., 2016).

The spatial description of the vertical organization of neurons in the cerebral cortex, cortical columnarity or minicolumns, is associated with psychiatric and neurological diseases. The organization of cells from layer III of Brodmann area 4 of the human cerebral cortex, studied with a homogeneous Poisson process, showed spatial randomness and a Poisson line cluster point process (Rafati et al., 2016). Randomness is also prevalent in the synaptic organization and composition of DCVs, which are controlled secretory organelles originating in neurons of the hippocampus. They contain proteins that mediate differentiation and synaptic plasticity. DCV dissemination along shafts, and inside synapses, followed Poisson statistics, demonstrating that the stochastic structure bears the cargo function. This type of a stochastic design supports the significance of DCVs localized to the synapse and extra synaptic sites (Robinson et al., 2016).

Microglia creates a network of tissue-resident cells associated with the resolution of CNS diseases. Microglial self-renewal within steady-state settings establishes a stochastic course. In the resolution phase of pathological conditions, the extra disease-associated microglia are removed by apoptosis to re-establish the steady microglial network (Tay et al., 2017).

Synapses undergo changes during plasticity due to variability in spike outlines, exhibited as sequential stochastic and spatial randomness. The variability arises from four features of spike configurations: synchronous firing, burstiness/regularity, heterogeneity of rates, and heterogeneity of cross-correlations. The upsurge in the decay time scale of the inhibitory synaptic flows, the integrate-and-fire network, is altered from an asynchronous to a weakly synchronous condition and then to a synchronous bursting condition. Burstiness is significant in regulating the variability of efficacy under asynchronous states, while the heterogeneity of cross-correlations is the major parameter determining the variability in efficacy when the network moves into synchronous bursting conditions, as for epilepsy (Robinson et al., 2016).

Electroencephalography (EEG) captures representative signals containing data relevant to brain function, and it is used to diagnose epilepsy. The prediction of epileptic seizures needs a detailed examination of the EEG recordings. EEG modeling can be used for automatic detection of epileptic seizures across different age groups, and detection of epileptic randomness with respect to the patient’s age (Hasan et al., 2017). The examination of EEG data shows developmental escalations in connectivity between distant brain areas. It demonstrates the reduction in randomness and increase in assimilation in brain networks, accompanied by a simultaneous increase in modularity. Minimum-spanning tree graphs were constructed by picking the strongest connections and avoiding loops; this resulted in a mainstay graph reflecting the qualitative properties of the network. Connectivity is found to increase from childhood to adolescence, and decreased from the age of 57 and over. An outline of increased integration and reduced randomness from childhood into adulthood was also demonstrated. Maturation at the neuronal level increased both connectivity and integration of the brain network (Smit et al., 2016).

Neural complexity is essential for maintaining consciousness. During loss of consciousness induced, for example, by injection of ketamine or propofol, this type of complex design is repressed. The randomness, which is termed type-I complexity, and complexity, termed type-II complexity, are analyzed from studies of EEG signals before and after these injections. The injection of each of the two drugs decreased the complexity of the signal recorded by the EEG, but ketamine augmented the randomness of the signal, while propofol reduced it. Hence, EEG complexity could be an indicator of consciousness (Wang et al., 2017).

The connectivity of the brain is continuously adjusted to environmental triggers via activity-dependent adaptive processes, such as synaptic plasticity, which controls the transmission efficacy of existing synapses. In addition, structural plasticity alters the connectivity by generating and deleting synapses (Fauth and Tetzlaff, 2016). In the nervous system, proper connections between neurons are sorted from an overwhelming number of potential but inappropriate connections. Models for the generation of synaptic specificity, which is based on the selection of nascent synapses according to adhesion and recognition, was proposed. This process is based on the highly dynamic and random nature of intracellular trafficking, and generates reproducible patterns of synaptic connectivity (Jontes and Phillips, 2006).

A recent review presented data on non-linear biomarkers for emotional disorders (anxiety, bipolar, and depressive), postulating that diseased systems display decreased complexity and flexibility with respect to adjustment to daily events. The results indicated that patients with anxiety present lower complexity than the control patients, and that patients who suffer from panic attack manifest irregular respiratory activity. Bipolar patients displayed increased randomness when suffering manic episodes. Patients suffering from depression demonstrated reduced complexity in the heart system and a decrease orderliness, even though they presented higher complexity in the brain. Overall, this study supports the hypothesis that patients with emotional disorders manifest with a loss of complexity or a loss of ordered complexity (de la Torre-Luque et al., 2016).

Functional near-infrared spectroscopy allows non-invasive continuous monitoring of alteration in blood volume and oxygenation in the brain. With this technique, it was demonstrated that the networks of people involved in spontaneous actions manifested increased clustering coefficients, smaller path lengths, increased mean node degrees, and profound randomness, in comparison to those of people involved in more controlled performances (Zhang et al., 2016).

### Randomness and Disease

Randomness may play a role in delaying the outbreak of infectious diseases (Muller and Koopmann, 2016). Gene regulatory networks were restored via differential equation models, to analyze networks in influenza subjects. Compared to asymptomatic patients, the symptomatic ones manifested increased amounts of driver nodes and intensity of critical links, and decreased amounts of redundant links. Furthermore, the stability of high-dimensional networks was sensitive to randomness in the reconstructed systems (Sun et al., 2016).

In dogs, it has been shown that statistical modeling of the variability of drug efficacy, in therapy for parasites, has an important contribution from the variability in parasite establishment rate. For example, heartworm “strains” alter their vulnerability phenotype over short time intervals, conveying that a large variety of vulnerability phenotypes occurs among naturally circulating biotypes (Vidyashankar et al., 2017).

Texture analysis of breast magnetic resonance images is used for identifying estrogen receptor-positive malignant subtypes. Properties which measure heterogeneity (smoothness) and homogeneity (randomness) reflect the underlying growth patterns of breast tumors (Holli-Helenius et al., 2017).

Modeling of woven bone microstructure as a composite – containing a milieu of mineral, collagen fibrils, and hydroxyapatite, in random orientation – was presented. A self-consistent homogenization scheme based on the randomness of the orientation of inclusions has been described (Garcia-Rodriguez and Martinez-Reina, 2017). Commercially available pure titanium and its alloys are used for bone replacement. Several models combine the simplicity of a 2D periodic geometric structure with the complex pore morphology in which the pores are circular and periodically distributed in the matrix, following a perfect pattern, or the pores are elliptical and have a randomly generated morphology, or the pores have a controlled random distribution determined by using randomness factors in both directions (Munoz et al., 2017).

Dose-response curves can be evaluated by two methods: a statistical method based on the presumed probability distribution of phenotypic parameters, and a deterministic method relying on the law of mass action. Chemical interactions between reacting molecules determine the dose-response curves of simple systems. The law of mass action reinforces these. The simple collision of molecules does not explain the dose-response curves of bioassays by a quantal response, but by phenotypic disparities among people. Individual tolerance is a random variable that results from genetic and environmental factors. The randomness of tolerance implies that deterministic equations are not required for such examination, and that statistical models are better tools for examining such dose-response relations (Mougabure-Cueto and Sfara, 2016).

A physiological lattice model for the passage and metabolism of drugs in liver has been described. The randomness of the 2D sinusoidal structure was found to give rise to variations in drug response in individual hepatic veins at different times. This model supports the fact that individual ports react in different ways, and that the typical liver lobule production over all the hepatic veins is more diffuse. The overall impact of these alterations is associated with a smoother drug response (Rezania et al., 2016).

### Randomness and the Human Psyche

In psychology, a steady-state visually evoked potential reflects a selective attention-based brain-computer interface (BCI) paradigm. The benefits of BCI are increased rate of data transfer, profound achievement across users, and increased tolerance to artifacts. It is associated with mental load and exhaustion while focusing on visual stimuli. Noise is a random perturbation which can be broken by the visual system to augment higher-level brain performances. A steady-state motion visual-evoked potential-based BCI paradigm, with spatiotemporal visual noise, was designed in accordance with this premise. It is used for measuring the consequences of noise on the response to mental load and fatigue, during tasks that require attention. A moderate level of visual noise was found to alleviate fatigue and mental load during the function of a visual BCI that required attention practices (Xie et al., 2017).

Coordination in groups can be improved by randomness. Studies using a networked color coordination game involving groups of humans that interact with autonomous bots have been conducted. The bots were programmed with variable degrees of randomness. Bots acting with minor degrees of random noise and placed in central locations improved the collective group performance. Behavioral randomness eased the task of humans to whom the bots were connected (Shirado and Christakis, 2017).

Studies of the tendencies followed in selecting and altering positions in the game of hide-and-seek were conducted for determining the effect of randomness on the selection. The creator of the game randomly used hidden numbers. Common tendencies were explored by comparing the number patterns in the human-generated calendars with random number patterns. The varying distance between the hidden numbers demonstrated an association between subjective randomness and the act of hiding (Sanderson, 2018).

### Randomness as a Tool for Improving Function of Systems

Non-linear mathematics, the complexity theory, and the chaos theory are often implemented in the analysis of dynamic biological systems. The concept of non-linear regulatory systems can function far from equilibrium conditions and that maintaining steadiness is not a mandatory goal of physiologic control under all conditions was proposed (Goldberger et al., 2002). Biological systems are and can be viewed as “complex adaptive organizations,” capable of adapting to environmental changes. It was proposed that health can be perceived as a continuous adaptation, while chronic illnesses represent a rigid dysfunction (Lipsitz and Goldberger, 1992; Lipsitz, 2002; Martinez-Lavin et al., 2008). The loss of complexity in diseased states is associated with an impaired ability to adapt to physiologic stress, and may underlie chronic diseases and aging (Kyriazis, 1991, 2003; Soloviev, 2001).

Biological systems do not always function under fixed rules which are constant over time. Biological systems are dynamic and multifactorial, this is in contrast to systems that may be described by physics, which contain parameters that are easier to control (Gsponer and Babu, 2009; Lodygin and Flugel, 2017; Buckle and Borg, 2018; de Lorenzo and Schmidt, 2018). The lack of rules in biological systems and the continuous dynamics while responding to trigger(s) may underlie part of the unpredictability of the response to drugs. The optimal state in variability may have a U shape between a chaotic pattern of variability in a steady state and full predictability in a normal biological system (Stergiou and Decker, 2011; Ilan,2019a,b,c,e,f). Dosing of medications using regular fixed regimens may not be compatible with the physiological variability in the immune system and may further contribute to loss of response (Weiner et al., 1980; Toni and Tidor, 2013; Ilan,2019c; Kenig and Ilan, 2019; Khoury and Ilan, 2019).

A dynamic nature of a system may be associated with its evolution into an assembly that optimizes its function (Orsini et al., 2015). Under certain conditions, promoting or making use of variability-associated parameters, may be necessary for improving the function of processes and systems (Ilan,2019f). It was also proposed to be use as a method for improving and/or correcting malfunction or disease states (El-Haj et al., 2019; Ilan,2019d, f; Kenig and Ilan, 2019; Khoury and Ilan, 2019).

Utilizing randomness in the treatment of every patient cannot be expected to produce the same effect in each case. Thus, utilizing randomness requires tailoring it to the specific patient and disease (Ilan,2019b,c,e,f). Enhancing the efficiency using patient-tailored approach requires increasing the degree of complexity of these systems (Gilra and Gerstner, 2017).

## Summary

Given that complex systems are different from each other, and their individuality is linked both to their composition, structural organization and environmental context in which they are embedded, and the way they respond to internal and external triggers, it is not surprising that their behavior sometimes unpredictable. Randomness may underlie several fundamental processes in many fields of life. Biological randomness and its potential role in ontogenesis and in evolutionary dynamics can be used as a method that contributes to the organism’s structural stability by adaptation and diversity (Calude and Longo, 2016; El-Haj et al., 2019; Ilan,2019b, c).

While it may seem somewhat paradoxical to the traditional dogma of order that is commonly accepted in physics, chemistry, and biology, the above examples suggest, that randomness may characterizes some of these systems under certain conditions. Furthermore, using or promoting the randomness may even improve the function of systems in some cases.

## Disclosure

YI is the founder of Oberon Sciences and consultant for Teva, ENZO, Protalix, Betalin Therapeutics, Immuron, SciM, Natural Shield, Tiziana Pharma, Plantylight, and Exalenz Bioscience.

## Author Contributions

YI analyzed the data and prepared the manuscript.

## Conflict of Interest

The authors declare that the research was conducted in the absence of any commercial or financial relationships that could be construed as a potential conflict of interest.

## Abbreviations

ApEn: approximate entropy
BCI: brain-computer interface
CHF: chronic heart failure
DCVs: dense-core vesicles
ECG: electrocardiogram
EEG: electroencephalographic
GA: genetic algorithm
HRV: heart rate variability
PVC: premature ventricular contraction
RN: random number
VIMs: variable importance measures.
